# Local reputation, local selection, and the leading eight norms

**DOI:** 10.1038/s41598-021-95130-3

**Published:** 2021-08-16

**Authors:** Shirsendu Podder, Simone Righi, Károly Takács

**Affiliations:** 1 Department of Computer Science, University College London, London, UK; 2 Department of Economics, Ca’Foscari University of Venice, Venice, Italy; 3 Department of Management and Engineering, The Institute for Analytical Sociology, Linköping University, Linköping, Sweden

**Keywords:** Evolution, Social evolution

## Abstract

Humans are capable of solving cooperation problems following social norms. Social norms dictate appropriate behaviour and judgement on others in response to their previous actions and reputation. Recently, the so-called *leading eight* norms have been identified from many potential social norms that can sustain cooperation through a reputation-based indirect reciprocity mechanism. Despite indirect reciprocity being claimed to extend direct reciprocity in larger populations where direct experiences cannot be accumulated, the success of social norms have been analysed in models with global information and evolution. This study is the first to analyse the leading eight norms with local information and evolution. We find that the leading eight are robust against selfish players within most scenarios and can maintain a high level of cooperation also with local information and evolution. In fact, local evolution sustains cooperation under a wider set of conditions than global evolution, while local reputation does not hinder cooperation compared to global reputation. Four of the leading eight norms that do not reward justified defection offer better chances for cooperation with quick evolution, reputation with noise, larger networks, and when unconditional defectors enter the population.

## Introduction

Cooperation between unrelated individuals in large scale societies is difficult to explain. Among humans, cooperation exists in a wide range of contexts including trade, joint work, and collaborations. Direct reciprocity explains cooperation in interactions that are likely repeated over time^1,2^. The *indirect reciprocity* paradigm claims that cooperation can also be viable when cooperation is not directly reciprocated by the interaction partner, but by a third party who has either observed or has been informed about the interaction^3,4^. For indirect reciprocity to be viable, the information on who has acted good and bad has to be passed on to future interaction partners.

What can be considered as good (and as bad) behaviour, however, is not evident. Indeed, while the existence of *social norms* is a universal feature of human societies, they are to a large extent culture-specific^3,5^. Norms guide behaviour (strategic response of cooperation or defection) and judgement of others (*reputation*) in light of previous actions and of previous judgements^6^. Because of the latter element, social norms are the tools of indirect reciprocity that pave the path for cooperation through *reputational dynamics*. The simplest social norm dictates cooperation if the opponent has a good reputation and assigns good reputation for cooperation. This is a binary version of image scoring^7^ according to which cooperative/selfish actions either increment/decrement a person’s image score. This social norm, however, does not allow the positive evaluation of defection under any circumstances, even if defection was directed towards an opponent who did not deserve help. Under image scoring, conditional cooperators are refused help if they themselves refused help to a non-cooperative individual. This is doubtful as humans have been proven to punish others even if it implies costs for themselves^8–14^ and this form of altruistic punishment could contribute to the evolution of cooperation^8,15,16^. Besides, while the image scoring norm promotes high levels of cooperation^7^, its stability on different population structures came into question^17^. Indeed, it was shown^18^ that cooperation under image scoring depends on very strong drift or a very small cost of giving help. Furthermore, in the presence of implementation or assignment error, theoretical work^19^ showed that image scoring is unable to sustain cooperation without an additional mechanism^20,21^, namely social network evolution.

To address the shortcoming of image scoring in the misclassification of justified defection, the standing social norm was shown to be superior to image scoring^18^. According to this social norm, individuals lose good standing by failing to help others in good standing, whereas withholding help from others in bad standing does not damage their standing^19,22^. Individuals without good standing can regain it by offering help to an individual with good standing. The main difference between the standing social norm and image scoring is that the standing social norm takes not only the action of the focal player but also the reputation of its opponent into account when making an evaluation, hence it is considered as a *second order social norm*. Adding further complexity^23,24^, *third order social norms* take into consideration the action and reputation of the focal player, as well as the reputation of the opponent for reputation update. Consequently, the behavioural strategies associated to social norms condition action to both own and opponents’ reputation.

**Table 1:** Payoff matrix for the Prisoner’s Dilemma (PD) game.

		B	
		C	D
A	**C**	(b–c, b–c)	(− c, b)
	**D**	(b, − c)	(0, 0)

We have $b=2$ and $c=1$. Payoffs are of the form (Payoff(A), Payoff(B)). If both A and B cooperate, they both pay a cost *c*, and gain a benefit *b* from the other’s cooperation. If only one of A or B cooperate, then the defecting agent pays no cost (while the cooperating agent does) and receives only the benefit *b*. If neither agent cooperates, then nothing is gained or lost.

**Table 2:**
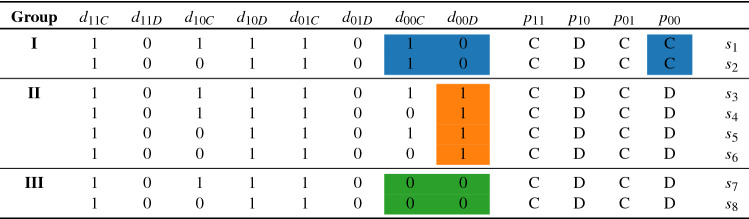
The *leading eight* social norms^23^.

The left eight columns contain the reputational update and the right four columns represent action response. An agent with reputation *m* interacting with another agent with reputation *n* will cooperate/defect according to $p_{mn}$. Similarly, the opposing agent will act according to $p_{nm}$. New reputations after interaction are assigned as $d_{mnp_{mn}}$ and $d_{nmp_{nm}}$ respectively.

In a hunt for social norms that could promote cooperation, from a total of 4096 possible third (or lower) order norms, only eight have been found to sustain cooperation and to be successful in monomorphic populations (a population consisting only of a single strategy) against an attack of unconditional defectors (AllD) or against a rare mutant of any alternative behavioral strategy^23,25^. In the setting considered, a large population of individuals engage in a one-shot helping game of the form of a simple two-person Prisoner’s Dilemma displayed in Table 1. Individuals are matched randomly in each round. The player who cooperates pays the cost of *c*, while the other player receives the benefit of $b>c$. For the cooperation decision, individuals rely on reputation assigned by social norms. It has been shown in an analysis of Evolutionary Stable Strategies (ESS) that these *leading eight* norms can promote a very high level of cooperation with an average payoff per game close to the maximum of $b-c$ even when errors are included in executing cooperation and in reporting the observation to the public and when the benefit of help only slightly exceeds its cost^25^. The leading eight social norms ($s_1 \ldots s_8$) are displayed in Table 2. Their joint characteristics are displayed in the columns with uniform values^23^. Concerning reputation update, the first column describes the *maintenance of cooperation*: assigning good reputation for cooperation between good actors. The second and the sixth columns represent the *identification of defectors* that imposes bad reputation if an actor refuses to cooperate with a good opponent. *Justified punishment* of bad opponents by good actors is expressed in the fourth column. *Forgiveness* is displayed in the fifth column: bad actors can gain a good reputation by cooperating with good actors. These joint characteristics highlight the mechanisms shared by norms that enable cooperation to be achieved through indirect reciprocity: (a) cooperation is maintained among cooperators; (b) when defectors enter, they are identified and labelled with bad reputation; (c) bad players are refused help, and those who refuse help to them are not sanctioned; (d) if a player—who has a bad reputation - “apologizes”, he will be forgiven^23^.

Differences between the leading eight norms can be found in the other three columns defining reputation update and in the prescribed action when two individuals of bad reputation interact. Based on these differences, the leading eight norms can be categorized into three groups^25^ (Table 2). The peculiarity of group I norms is to cooperate in an interaction of individuals with bad reputation. Group II norms are characterized by justified defection towards an opponent of bad reputation even if they themselves had no good reputation^18,19,26–29^. Finally, norms in group III are the strictest as they prescribe defection against an individual with bad reputation and keep the bad reputation of the focal player after any choice towards an opponent with bad reputation. These differences among the leading eight norms, however, do not seem to matter for the maintenance of cooperation^23,25^.

These results could be misleading, however, as the success of social norms have been analysed in models with global information and evolution. This is somewhat awkward as indirect reciprocity has been claimed to extend direct reciprocity in larger populations where direct experiences cannot be accumulated^3^. To shift the focus of investigation of social norms that can sustain cooperation towards a more realistic ground in this aspect, our aim in this study is to analyse the leading eight norms with local information and evolution.

Once one of the assumptions of well mixed populations, globally available reputations, and global strategy update is relaxed, the network structures on which evolutionary games are played become important^30,31^. Previous research found interesting results about the evolution of cooperation in sufficiently sparse networks^32,33^, small-world topologies^34^, and in other realistic network structures^35^, but also when networks evolve, either through non-random partner selection^36^ or self-organization of social/emotional ties^37–39^. The majority of literature examines networks in which edges denote the pairs of players who are allowed to interact. For indirect reciprocity, however, ties that enable the flow of information and ties that are used to learn strategies from others are crucial. Even if interaction could take place between any two individuals and decisions are assisted by information attained via network relations.

**Figure 1:**
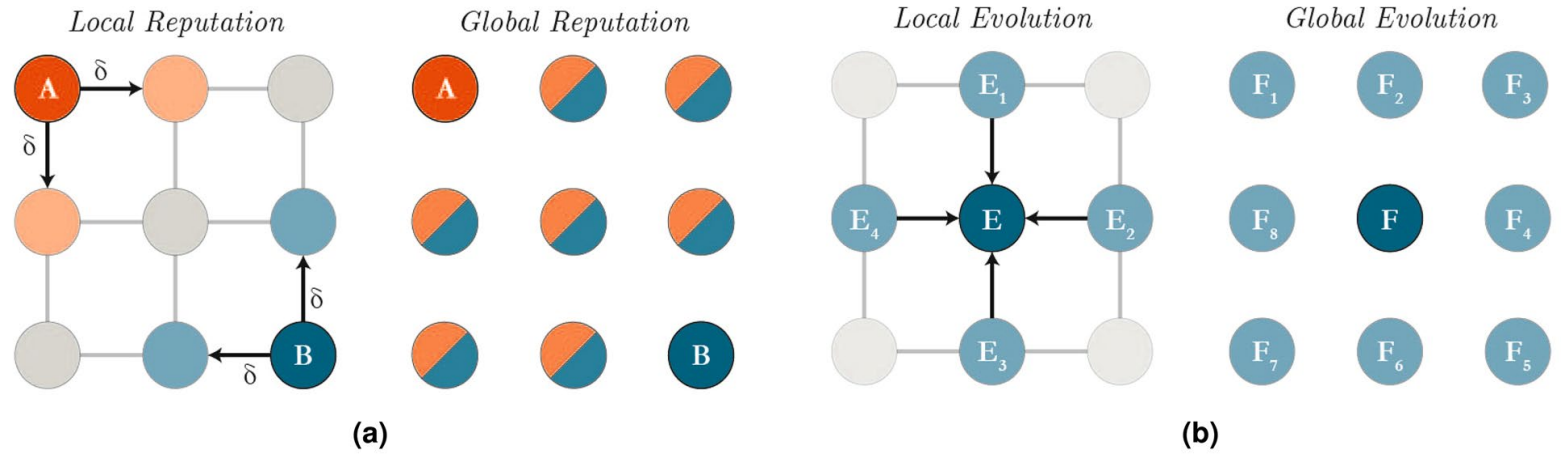
Reputation and evolution mechanisms. (**a**) Suppose agent *A* and *B* are chosen to interact with each other. With *local reputation*, *B* can only ascertain *A*'s reputation from *A*'s neighbours. Likewise, *A* can only ascertain *B*'s reputation from *B*'s neighbours. These neighbours have probability δ of witnessing each of *A* and *B*'s interactions and their most recent reputation. This is in contrast to *global reputation* where an agent's reputation is publicly known information. (**b**) With *local evolution*, agent *E* can only view the payoff (and the corresponding strategies) of his neighbours *E*_1,...,4_ and can update to the strategy that results in the greatest payoff in his neighbourhood with probability α. With *global evolution,* agent F can analyse the entire population, in this case *F*_1,...,8,_ and find the strategy with the greatest average payoff amongst agent running that strategy.

The contrast between local and global evolution and between local and global reputation update are illustrated in Fig. 1. In most contexts, individuals are unlikely to have a perfect view on the most beneficial strategies in the entire population. Accordingly, local evolution models situations in which only success in the network neighbourhood is considered. Similarly, global reputation allows individual reputation to be common knowledge. Considering local reputation update, an individual *A* who is not directly connected to opponent *B* needs instead to query a neighbour of opponent *B* to ascertain *B*’s reputation. This implementation reflects that *B*’s direct peers witness the reputation of *B* with probability $\delta$. Using these local sources of information and the social norm they follow, both *A* and *B* can appropriate their decision and are then subsequently judged for it. For reputation to be effective, it needs to be available to future interaction partners as reputation is used to transmit information in the absence or in addition to direct observations. For these reasons, reputation is never completely “local” when interactions are global. In our model, reputation is local in the sense that it can be accessed only through the focal agent’s neighbours. Local reputation update is noisy in the sense that it can be learnt with probability $\delta$ and is possibly incorrect with probability $\nu$.

In this study, we explore if the leading eight social norms could still support cooperation considering local evolution and reputation update. By introducing unconditional defectors into a homogenous population of players who all follow a leading eight norm, we investigate the constraints that ensure the survival, or conversely, the extinction of the leading social norm. We compare the extent to which cooperation can be sustained in all combinations of local and global reputation (see Fig. 1a) and evolutionary (see Fig. 1b) updates (Table 3). We also compare the performance of leading eight norms in groups I, II, and III considering global and local evolution and reputation. Furthermore, we analyse the robustness of cooperation by social norms under each regime manipulating the speed of evolution $\alpha$, the likelihood of errors in reputation broadcast $\delta$, the intrusion probability of AllD $\beta$, the initial proportion of free riders, and varying the network structure, density, and population size.

**Table 3:** General overview of model variants. G and L represent Global and Local, r and e represent Reputation and Evolution.

		Evolution	
		Global	Local
Reputation	Global	GrGe	GrLe
	Local	LrGe	LrLe

## Results

To analyze our model, described in detail in the “Methods” section, we assess the individual effects of the main parameters under all four combinations of local and global strategy update and reputation diffusion (Table 3). In each analysis, we focus on the final level of cooperation, averaged over 100 simulations for each parameter combination. We cluster results for each group of strategies, given that their behaviour is very similar to each other.

Our findings show that the *leading eight* norms—in a wide range of circumstances—maintain high levels of cooperation in Erdős–Rényi random networks against unconditional defectors. When populations have access to complete, errorless information (in global reputation networks or local reputation networks with $\delta =1$ and ν=0), all leading eight norms maintain very high levels of cooperation when initialised in homogeneous populations with unconditional defectors being introduced as mutants roughly once every ten time-steps.

**Figure 2:**
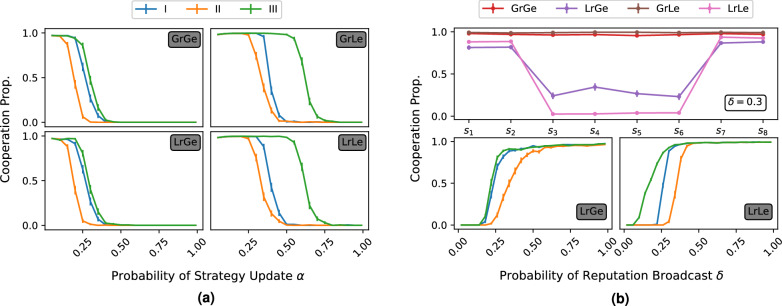
The effect of the speed of evolution and reliability of information on cooperation. (**a**) The quicker the evolution, the greater the domination of unconditional defectors. Under global evolution (left panels) we can clearly see that group II norms require a slower speed of evolution than either groups I or III to resist unconditional defectors. Here, groups I and III display very similar behaviour declining at an almost identical rate. This holds true for both global and local reputation. Under local evolution (right panels), group III norms can maintain cooperation under the widest range of speed of evolution. In all simulations, δ = 1 (perfect broadcast of reputation). (**b**) The impact of unbiased transmission of reputation on cooperation. The upper panel shows the case in which group II fails in maintaining cooperation in the population under local reputation (δ = 0.3). Here LrLe is fully invaded by unconditional defectors, whilst there is between 25-35% cooperation in LrGe (see Supplementary Fig. S6). The lower panels display the effect of imperfect information through local reputation transfer. When δ = 0, players guess their neighbour’s reputations, and when δ = 1, they retain perfect knowledge of their neighbours social standing. Note that in our model, δ has no effect on global reputation. We see in LrLe that the harshness of group III towards defectors is particularly suited in uncertain environments. Group II norms are consistently the most sensitive towards imperfect information. In all simulations, α = 0.1 a value that, in Fig 2a, always enables full cooperation.

**Figure 3:**
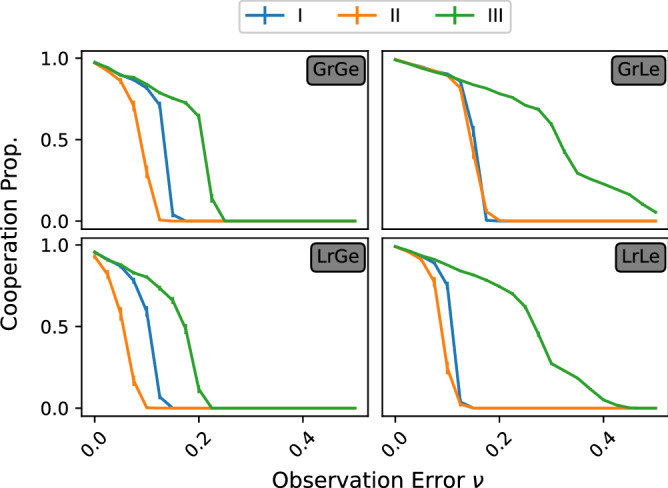
Effect of observation error $\nu$ on the proportion of cooperation. Global reputation provides a slight advantage over local reputation. Local learning shows increased resilience against more likely errors in observation mostly for group III strategies. Additionally, there is little to distinguish between the behaviour of groups I and II under local learning, particularly in combination with global reputation.

When reputation broadcast is weakened (lower panel of Fig. 2b), we see that all three groups of strategies eventually fail^19^. Group II norms are the most dependent on the accuracy of reputational information. Within model LrGe , when $\delta < 0.25$ (when less than a quarter of an agent’s neighbours witness his interaction and are aware of his new reputation), we observe that group II norms fail to maintain cooperation. When $0.25< \delta < 0.4$, we see a steep increase in cooperation until $\delta > 0.4$, at which point we have almost full cooperation. Groups I and III display almost identical behaviour, being able to withstand lower $\delta$s yet maintaining high levels of cooperation. Thus, within the LrGe model, group II requires more accurate information to sustain cooperation than groups I and III.

In comparison, in the LrLe model, we observe a divergence of behaviour between groups I and III. Group II is again the weakest of the three, requiring more accurate information than the others to maintain cooperation. Moreover, whereas groups I and III were very similar in LrGe, here we see that group III strategies have a much lower $\delta$ threshold for cooperation than group I. Thus, within LrLe, there is a clear hierarchy of social norms; group III is the most resilient towards information scarcity, followed by group I, and then by group II.

Directly comparing the evolutionary mechanisms in Figs. 2b and 3 and in Supplementary Figs. S7 and S8, local evolution of strategies seems to provide better conditions for cooperation than global evolution, but consistently only for group III. When we allow cognitive errors in the evaluation of reputations (Supplementary Fig. S8), the benefits of local evolution become significant only for group III. Under global learning, reputation based on local observations is at best just as good as global observations, while learning locally allows consistently better cooperation when individuals use reputation formed from observations that are local rather than global. In the absence of cognitive errors (Fig. S7), while the improvement is mostly marginal, the main benefit comes when the likelihood of witnessing an event is quite low with $\delta \in [0.05, 0.25]$, where we see an increase of up to 40% over global evolution. For groups I and II, we also see a marginal increase of cooperation for worlds in which communication is less uncertain and more timely. When $\delta > 0.3$ and $\delta >0.38$ for groups I and II respectively, similarly to group III, we observe minimal increases in cooperation of local evolution over global evolution. When accurate and timely information becomes scarce, we see that for both groups I and II, global evolution provides better conditions for cooperation. Group I strategies have $d_{00C}=1$ and $p_{00}=C$ which make them particularly vulnerable when engaging with AllD, especially when there is so much uncertainty concerning an opponent’s reputation.

The same hierarchical pattern between the groups is found when considering the speed of evolution (in Fig. 2a), parameterised by $\alpha$. A greater speed of evolution has been previously associated to better outcomes for defection^38,40^. The same pattern of behaviour emerges here. The resilience of social norms to a more dynamic evolutionary environment resembles the one determined by information scarcity. Indeed, throughout each model, group III strategies maintain greater cooperation than the other groups. Again, with global evolution, we see that groups I and III behave very similarly. Under local evolution, group III can withstand much more rapid evolution than other norms before defection becomes prevalent.

**Figure 4:**
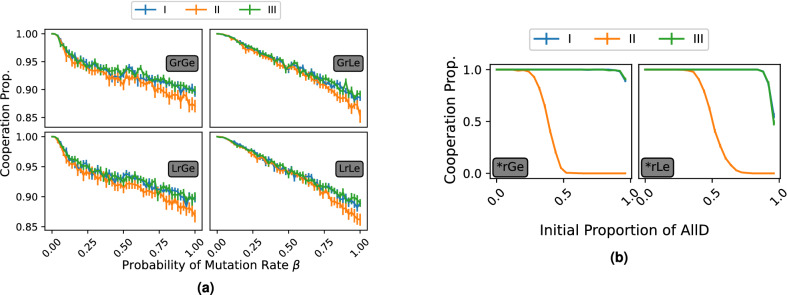
The effect of the likelihood of an AllD mutant entry and the initial proportion of AllD on cooperation. (**a**) As the rate at which AllD agents are introduced into the population increases, the level of cooperation decreases. Under local evolution, we see that the decrease is generally linear. Under global evolution, after the initial drop in cooperation for small β values, for 0.15 < β < 1 we see a smooth decline in cooperation as the rate of mutations increases. (**b**) The differential impact of the initial proportion of AllD agents on the success of social norms in maintaining universal cooperation. Group II strategies lose their dominance starting from 25% of AllD players at the outset in global evolution (left) and from 40% in local evolution (right). In contrast, groups I and III maintain their dominance even if a large majority of the population initially consist of AllD agents.

Another way in which it is possible to study the resilience of a social norm is to assess its robustness against invasion by mutants. We approach mutation in two ways. First, a mutant of universal defection (AllD) can enter in each step throughout the simulations with some probability $\beta$ (in Fig. 4a). Second, some initial proportion of the population is set to be AllD (in Fig. 4b). When $0< \beta < 1$ (up to a single AllD mutation in each time-step on average) we see generally the same monotonically decreasing level in cooperation within the population. While we observe a similar pattern in Fig. 4a in all four model environments, when comparing them against increasing AllD proportions of the initial population, we see that groups I and III can still force cooperation to a similarly large extent within the population despite being scarcely represented at the outset. At the same time, group II norms are only resistant to a lower initial proportion of AllD players in the population.

In general, all social norms eventually fail on Erdős–Rényi networks when conditions become harsher, in a typical order of groups II, I, and III being the most resistant of all. Using a baseline of a single AllD mutation once every ten time-steps (on average), we conducted a sensitivity analysis (reported in Supplementary Figs. S2–S13) on the parameters of each network type: Regular Random Lattices (RRL)—parameterised by *d* representing the number of neighbours for each agent, scale-free (SF)—parameterised by the Barabási-Albert preferential attachment parameter *m*, and Watts–Strogatz Small World (WSSW) networks—parameterised by the initial degree *k* and the probability of rewiring *p*. In each network, for each of our models, we see that our main conclusions hold with no significant deviations, and large-scale cooperation can be maintained by the leading eight social norms.

Additionally, simulations were run increasing the size of the Erdős–Rényi network and the total simulation time (in Supplementary Figs. S14 and S15). The globally evolving populations exhibit lower cooperation for each group of strategies as the number of agents increase, particularly affecting group II. Locally evolving populations, however, can maintain stable cooperation until the population consists of at least 800 agents , the largest population we explored systematically.

Unlike the original leading eight analysis^25^, in this paper we focused on the ability of leading eight norms to preserve cooperation when invaded by AllD players, thus concentrating on the most immediate threat to cooperation, and not on the more general concept of Evolutionary Stable Strategy. Unlike AllD, more benevolent invaders could be indistinguishable from the incumbent norm, thus leading to the long run persistence of mixed populations. We report these and further results on the invasion by AllC in the SI, Figs S9–S11.

## Discussion

Cooperation is of paramount importance for the functioning of human societies. Why people cooperate to a large extent with strangers whom they have no direct experience with is a difficult puzzle^32^. Indirect reciprocity offers a possible solution through the assistance of social norms that guide individuals to distinguish good opponents from bad ones and prescribe appropriate action^3,18,41,42^. From many potential candidates, previous research has identified the *leading eight* norms that can sustain large scale cooperation when information is publicly shared^23,25^. The assumption that reputational information is publicly and unbiasedly shared, however, is questionable^40,43,44^. Humans, if they wish to condition their action towards others appropriately, must rely on direct observation or on reputational information that originate from network contacts who can judge the individual accurately. Moreover, selection takes place locally and not at the global scale. In this study, we have considered these two fundamental features of local embeddedness for the analysis of social norms that could establish and maintain large scale cooperation.

Our major finding is that when $\alpha$ (the likelihood of strategy update) and $\nu$ (the probability of observation error) are sufficiently low and $\delta$ (the probability of reputation broadcast) is sufficiently high, the leading eight social norms are able to sustain cooperation also with locally shared reputation and local selection pressure. Under these conditions, a world in which interactions are not witnessed by all individuals does not provide largely worse or better conditions for social norms to maintain cooperation than a world in which reputations are publicly shared. The probability of reputation transfer plays a crucial role in determining the ability of social norms to sustain cooperation under local information sharing. We found almost no cooperation with low probabilities ($\delta <0.3$), confirming results of earlier work on indirect reciprocity with private reputations^43^. Importantly, in a middle range ($0.3\le \delta <0.4$), the ability of the leading eight strategies to maintain cooperation is differentiated: groups I and III are able to support cooperation, while group II that is characterized by rewarding justified defection is not. For higher probabilities of reputation transfer, we found marginal increases in coopertation with higher $\delta$s (Supplementary Fig. S6).

The local evolution of behavioural strategies seems to provide better conditions for cooperation than global evolution, except when considering Group II norms under certain specific parameter conditions (see Fig. 4b). For moderate to high values of $\delta$, we see that local evolution improves cooperation because it slows down the transmission of AllD in the population. Suppose that agent *Z* is free-riding and that his behaviour is particularly fruitful in a sparse network where only his neighbours—a very small subset of the population—are aware of this high payoff and will adopt the selfish AllD strategy with some probability. As the density of the network (defined as $\lambda$, the probability of two edges being connected in the Erdös Rényi random network) increases, the subset of agents in the network witnessing *Z*’s success rises, increasing the number of players potentially adopting a more selfish strategy within any single evolutionary step. Hence, low density and local evolution are able to slow down the proliferation of universal defection and provide favourable conditions for large scale cooperation. Furthermore, for a given network, if the reputation broadcasting is sufficiently inefficient with respect to the speed of evolution, defection yields on average higher payoffs. There is always some probability, however (and this probability is higher for higher $\delta$s and lower $\alpha$s), that unconditional defectors are recognized and socially punished by the leading eight norm who refuses helping them. In these latter cases, AllD players obtain individually low payoffs, hence they are replaced by the leading eight strategists. In global evolution, what matters is the relationship between the two average payoffs, which means that single AllD players are protected by their better average performance. Local evolution provides better conditions than global evolution for cooperation also for a wider range of parameter values concerning the speed of evolution ($\alpha$), the probability of reputation broadcast ($\delta$), the probability of cognitive error ($\nu$) and the initial proportion of AllD players.

Another main difference between global and local evolution regimes is that the success of the leading eight norms considering local selection is more differentiated. We have shown that the leading eight maintain high levels of cooperation when agents are arranged on networks using global or local reputation and evolution. They resist unconditional defectors when there is no error in information transmission. When faced with error, extreme initial proportions of unconditional defectors within the population, faster rates of evolution, or larger networks, we find an inherent weakness of group II strategies.

Group II social norms are characterised by rewarding justified defection ($d_{00D}=1$)^45^, where two disreputable interacting agents are both rewarded for defection by an improvement in their social standing^18,29,46,47^. Justified defection has been under scrutiny in empirical research and there is only mixed evidence about its relevance in human decision making^44,48,49^. For group II norms, defection against disreputable players is considered good behaviour as $d_{10D}=1$ and $d_{00D}=1$. The former is a property of all leading eight norms and rewards the punishment of AllD entrants by individuals of good standing. Due to this property, AllD entrants are ignored by generous players. When an AllD player meets another universal defector by chance, they both defect. With group II social norms, however, they are both rewarded with a positive reputation, which leaves them free to ‘fool’ a player with cooperative intent the subsequent time. Repeating this process makes it more likely that AllD players collude amongst one another in the population. Because of these dynamics, group II norms are more vulnerable to larger initial proportions of unconditional defectors within the population, to larger mutation rates, and to error in reputation transmission than group I and III norms. The results also suggest that there may exist some threshold of the number of interactions between disreputable persons at which the spread and domination of AllD becomes irreversible. In Fig. 4b, this threshold under global evolution becomes apparent when 25-50% of the population consists of AllD strategists. For populations utilising local evolution, this threshold is significantly higher, occurring between 40-70%. Therefore we see that populations engaging in local evolution can withstand a greater amount of collusion amongst defectors than populations utilising global evolution.

The social norms of groups I and III do not allow the collusion amongst defectors as $d_{00D}=0$. While they seem to be superior performers to norms with justified defection considering many dimensions, the speed of evolution and mistakes in the reputation transmission creates a difference also between them (Fig. 2). Group III norms can generally maintain higher levels of cooperation than group I norms, which in turn is more likely to sustain overall cooperation than group II norms. The difference between group I and III norms can be attributed to the combination of reputation update $d_{00C}$ and behavioural strategy $p_{00}$. It is due to a forgiving characteristic of group I norms ($d_{00C}=1$ and $p_{00}=C$) that make them vulnerable to being taken advantage of by unconditional defectors. If they lose their good standing by mistake, they will be inclined to cooperate with bad players, which would allow them to regain their good reputation for other group I players. Partly this is for good purpose, as the opponent could also be in the same shoes. But the opponent could also simply be an AllD player who is reaping the benefits from the situation. By the same reasoning as with AllD against group II strategies albeit to a lesser extent, AllD strategists benefit from interacting with group I players who coincidentally lost, and would like to (and will) regain their good standing by cooperating with them. Considering group I norms, the reputation of AllD players becomes less important than the number of interactions they participate in. The greater the number of interactions, the greater the probability they dupe someone looking to regain their good reputation.

So far, we have seen that the subsets of interactions that are beneficial towards the spread of AllD shrink as we move from group II to I, and now to III. Within group III, there is no possibility of disreputable players regaining their social standing by cooperating against anyone ($p_{00}=D$ and $d_{00D}=0$) but another good person ($p_{01}=C$ and $d_{01C}=1$). Here, the only situations that may benefit an AllD player are the first interactions after mutation when the player still has a good reputation as testified by each of his neighbours or when the AllD player is known to have most recently defected, but is mistakenly thought to be good by a neighbour who has not witnessed or has misperceived his most recent interaction. These circumstances in which AllD can spread its influence are rare, causing group III norms to be the most robust of all. The relative cruelty in handling observed defection and bad reputation causes group III norms to be more resilient also towards errors in observation. In cases of larger uncertainty, they are better off being on the safe side and defecting against an individual with bad reputation while condemning a player after any choice towards an opponent with bad reputation. In times of observational uncertainties, being less forgiving seems to be beneficial when engaging in conditional cooperation.

We have conducted several robustness checks to support our main conclusions. We have varied the network structure and the density of the interaction network. Modifying the density of the Erdős–Rényi network continues to show the inherent relative weakness of rewarding justified defection (group II norms). In general, under local evolution, as population density increases, the level of cooperation decreases. Our results hold when alternative network topologies are considered, namely Random Regular Lattices (RRL) in Supplementary Figs. S2 and S3, Scale Free (SF) in Fig. S4, and Watts–Strogatz Small World Networks (WSSW) in Fig. S5. In all simulations of SF and WSSW networks, we see high levels of cooperation with no significant differences between them and Erdős–Rényi random networks except for generally higher levels of cooperation found in locally evolving populations.

The main real-world implication of our study concerns the effects of restricting and enhancing the mechanisms through which people acquire information about their peers’ reputation and through which they learn about their strategies. Today, online social networks increase the number of people we have access to (thus making interactions more “global”), but at the same time they weaken the transfer of information about individual reputations (increasing the noisiness of reputation transfer), allowing cheaters to build positive reputations that can then be used to exploit or mislead a large mass of individuals. Examples of such behaviours are the malicious spread of fake news and of online fraud. Our study suggests that the introduction of effective and reliable reputational mechanisms is key in supporting the persistence of good behaviour, and in limiting the spread of anti-social behaviour. In the impossibility of having a perfect reputational system for localised interactions, our study further suggests which of the good reputational norms work best under a wide range of conditions. These norms are harsher towards defection and where—once lost—good standing is difficult to be rebuilt. Finally, our results suggest that where communication of reputational information is poor, mechanisms inducing individuals to copy the successful behaviour of their peers are better suited to support cooperative behaviour than learning from globally established information.

As a limitation of our study, we note that our comparison between social norms is not a strict one-to-one comparison with the conditions derived^23^ for the stability of each social norm against AllD. Our analysis demonstrates the resilience against AllD in a more dynamic setting, both in global and local evolution. Our implementation exclusively uses 3rd-party opinion for reputational information. This interpretation of reputation is more realistic and justifies also the need to consider social norms, the transmission of reputation information, and strategy updates in the local rather than in the global context.

Subsequent studies may tackle the relative strength of social norms further by considering heterogeneous populations^50^ situated on networks. Furthermore, the consideration of parallel existence of conflicting social norms in the population^51^ widens the questions on how post-interaction reputations are assigned by different groups of people exhibiting different interpretations of good behaviour. It is an increasingly complex question how cooperation could evolve when the population is largely divided on the question of what is considered to be good and bad behaviour.

## Methods

Consider a static and connected graph of *N* agents. In line with Ohtsuki and Iwasa^25^, we assign the population a single reputational update rule $d_i$ as well as a behavioural strategy $p_i$ to each of its players. The $(d_i, p_i)$ pairs constitute social norm $s_i$ where $i=1, \ldots , 8$ represent the *leading eight* and $i=9$ represent unconditional defectors.

We initiate the population with *kN* agents under $s_{i^*}$ for $k \in (0,1]$, and the remaining $(1-k)N$ agents as $s_9$ unconditional defectors. To start with, agents are assigned a good or bad reputation at random.

We begin the simulations with randomly generating a network with some structure (Erdős–Rényi random network in the baseline; extensions with regular lattices, scale-free networks, Watts–Strogatz small world networks) with the limiting requirement that every agent within the network must have at least 2 neighbours (to illustrate this, suppose there are two neighbouring agents *A* and *B* where $deg(A)>1$ and $deg(B)=1$. Here, agent *A* has no source of information about agent *B* except himself. We forbid agents from using their own information to isolate the properties of 3rd-party opinion within our model).

Next, we simulate a maximum of $T_{max}$ time-steps. Within every time-step, we have a minimum of one interaction. Two agents A and B are randomly selected from the population to play the Prisoners’ Dilemma (PD) with the payoffs displayed in Table 1 wherein they cooperate or defect according to their own behavioural strategy and are judged upon their choice under the rules of the social norm. New reputations are assigned, payoffs awarded, and we select again two agents to continue with probability $\Omega$.

Once a time step ends, agents can update to a more beneficial strategy ($\{s_{i^*}, s_9\}$) with some probability. Under local evolution, this is implemented as a “copy-the-best” update rule where players update to the locally best strategy (the strategy of the agent(s) in the neighbourhood $F_i$ of agent *i* with the highest total payoff in that round) with probability $\alpha$. Under global evolution, each player *j* updates his strategy with probability $\alpha \cdot \tfrac{|u(A)-u(B)|}{u(A)+u(B)}$ where *A* is the globally better performing strategy and $u(X)=\max (\text {Average Payoff of Strategy X},0)$. Here, evolution does not allow players to move to anything but a better strategy, hence negative utilities are considered to be 0. In both mechanisms, $\alpha$ represents the speed of evolution. During each evolutionary step, each player randomly alters its strategy to AllD with probability $\frac{\beta }{N}$ that we label as mutation.

Convergence can be achieved in one of two ways. Either $T_{max}$ is reached or when the distribution of strategies in the population becomes approximately equal to the population in two randomly chosen, uniformly distributed prior time-steps. Convergence is prohibited in the first quarter of the simulation to allow the population a chance to evolve.

We carry out two main manipulations: the localisation of reputation (Fig. 1a) and of evolution mechanisms^23^ (Fig. 1b). Under global reputation, an agent’s reputation is public knowledge. Under local reputation, an agent’s reputation is only known to his neighbours. These neighbours witness the agent’s interactions (and therefore his new reputation) with probability $\delta$. Once witnessed, the agent’s interaction is interpreted incorrectly with probability $\nu$. When opponents interact with the agent, they first randomly select a neighbour (excluding themselves in the case they are directly connected), and use their evaluation of the agent as a guideline to select the appropriate action in the PD. In case where the focal agent has no previous interaction, his reputation is either good or bad with equal probability. An agent’s knowledge of his neighbour’s reputation is kept between time-steps.

## Supplementary Information

Supplementary Information.
