# Supplementary material for: Local reputation, local selection, and the leading eight norms
Source: Sci Rep. 2021 Aug 16;11:16560. doi: 10.1038/s41598-021-95130-3 (PMC8368190; doi:10.1038/s41598-021-95130-3)
Supplement: Supplementary file 1 — Supplementary Information. [file 41598_2021_95130_MOESM1_ESM.pdf]

Local Reputation, Local Selection, and the Leading Eight Norms: Supplementary Information

Shirsendu Podder[1,*], **Simone Righi**[2], **and Károly Takács**[3]

[1]Department of Computer Science, University College London, United Kingdom
[2]Department of Economics, Ca'Foscari University of Venice, Italy
[3]Department of Management and Engineering, The Institute for Analytical Sociology, Linköping University, Sweden
[*]Corresponding Author: s.podder@live.co.uk

ABSTRACT

Humans are capable of solving cooperation problems following social norms. Social norms dictate appropriate behaviour and judgement on others in response to their previous actions and reputation. Recently, the so-called *leading eight* norms have been identified from many potential social norms that can sustain cooperation through a reputation-based indirect reciprocity mechanism. Despite indirect reciprocity being claimed to extend direct reciprocity in larger populations where direct experiences cannot be accumulated, the success of social norms have been analysed in models with global information and evolution. This study is the first to analyse the leading eight norms with local information and evolution. We find that the leading eight are robust against selfish players within most scenarios and can maintain a high level of cooperation also with local information and evolution. In fact, local evolution sustains cooperation under a wider set of conditions than global evolution, while local reputation does not hinder cooperation compared to global reputation. Four of the leading eight norms that do not reward justified defection offer better chances for cooperation with quick evolution, reputation with noise, larger networks, and when unconditional defectors enter the population.

Contents

1 Network Explorations **2**
1.1 Erdös Rényi Random Network . . . 2
1.2 Heterogeneity . . . 3
1.3 Barabási-Albert Model . . . 5
1.4 Watts-Strogatz Small-World Networks . . . 6

2 Robustness Checks **7**
2.1 Probability of Reputation Transfer against AllD . . . 7
2.2 Local Evolution excess cooperation against probability of reputation transfer . . . 8
2.3 Observation error organised by group . . . 9
2.4 The Leading Eight against AllC . . . 10
Probability of reputation transfer against AllC • Speed of strategy update with AllC • AllC cannot invade the leading eight with local learning
2.5 Extreme Mutation Rate . . . 15
2.6 Simulation Length . . . 16
2.7 Size of network . . . 17

3 Success of groups I and III in AllD populations **18**

1 Network Explorations

1.1 Erdös Rényi Random Network

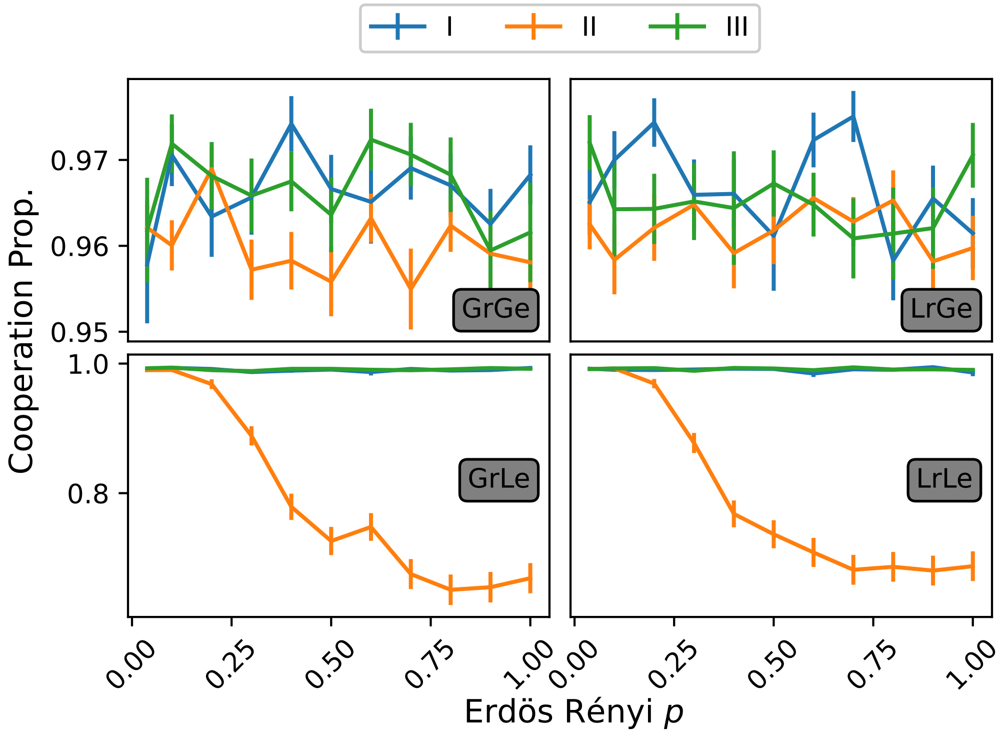

Figure S1. Effect of density on cooperation. Increasing the density of the Erdös Rényi random network has little effect on populations utilising global evolution. However, due to the combination of social norm $d_{00D} = 1$ and behavioural strategy $p_{00} = D$, group II is particularly susceptible to invasion from groups of unconditional defectors in populations adopting local evolution. We see that as the density of the network increases, the size of an unconditional defector's immediate neighbourhood also increases. This expands the number of neighbourhoods in which the unconditional defector's strategy is optimal, thus propagating the non-cooperative strategy further. An equilibrium is reached between unconditional defectors and group II strategies when the network is fully connected allowing for 60-65% cooperation. In all scenarios, strategies of groups I and III are generally unaffected, remaining stable with close to complete cooperation.

1.2 Heterogeneity

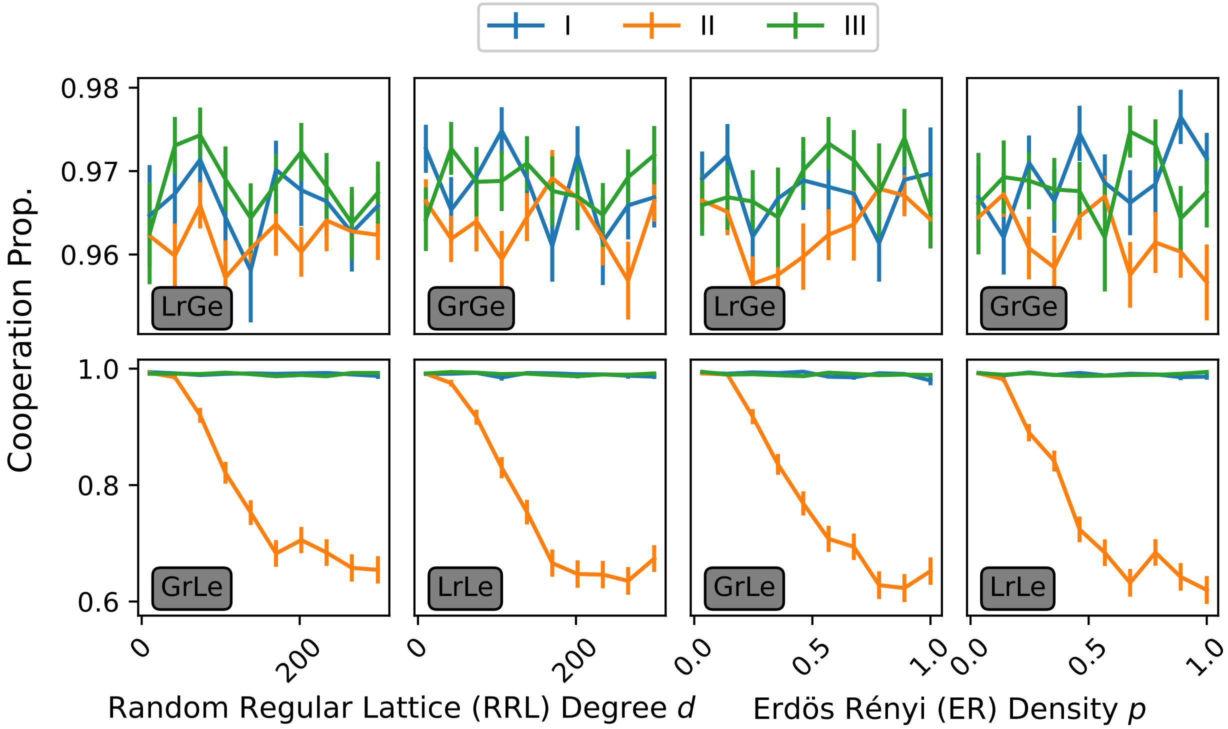

Figure S2. Effect of Heterogeneity on cooperation (macro). As with the ER random networks in Fig. S1, for the extreme values of degree d within the RRL networks, the weakness of group II is evident in populations with local evolution. With consistent findings between these network topologies, we see no discernible and unique relationship between degree heterogeneity and cooperation.

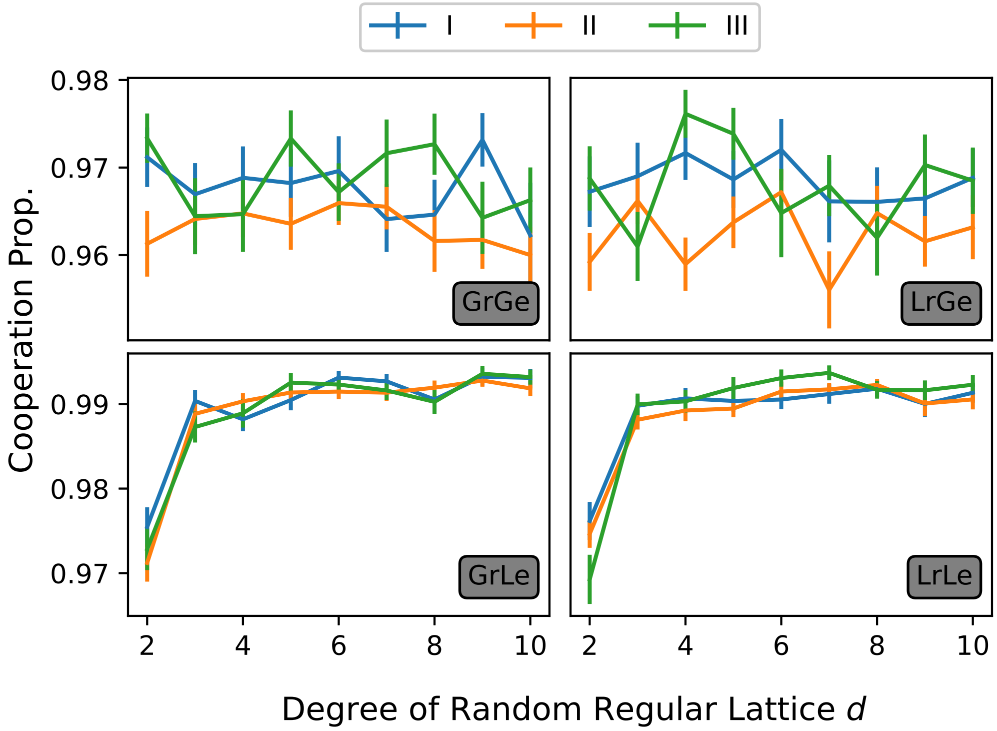

Figure S3. Effect of Heterogeneity on cooperation (micro). There is evidence of consistently high levels of cooperation independent of the particular choice of reputation and evolution. There are slightly lower levels of cooperation for $d = 2$ as this is a ring-lattice where each defecting agent can highly influence his two neighbours depending on α. With local evolution, we see a consistently lower standard error than populations with global evolution.

1.3 Barabási-Albert Model

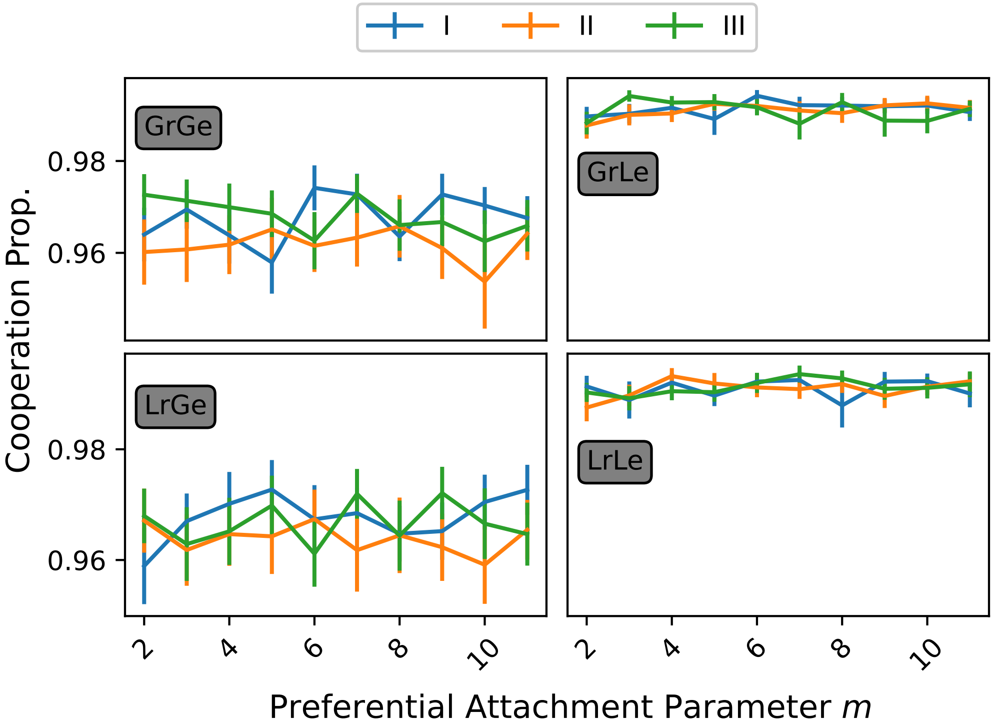

Figure S4. Consistently high levels of cooperation in Scale-Free networks varying the preferential attachment parameter. The Barabási-Albert model was used for the generation of random networks exhibiting scale-free properties. We see that there is little correlation between the preferential attachment parameter and the proportion of cooperative actions within the final time-step of the simulation. It may be expected that increasing the number of connections exhibits the same behaviour as the density of the Erdös Rényi network in Fig. S1, however this may still be the case as $m = 11$ is equivalent to a density of approximately 0.07. Therefore, even in an Erdös Rényi network, we would expect the same behaviour as witnessed here. We can again see the general benefit of local evolution over global as we can consistently see a marginally higher proportion of cooperation within the network.

1.4 Watts-Strogatz Small-World Networks

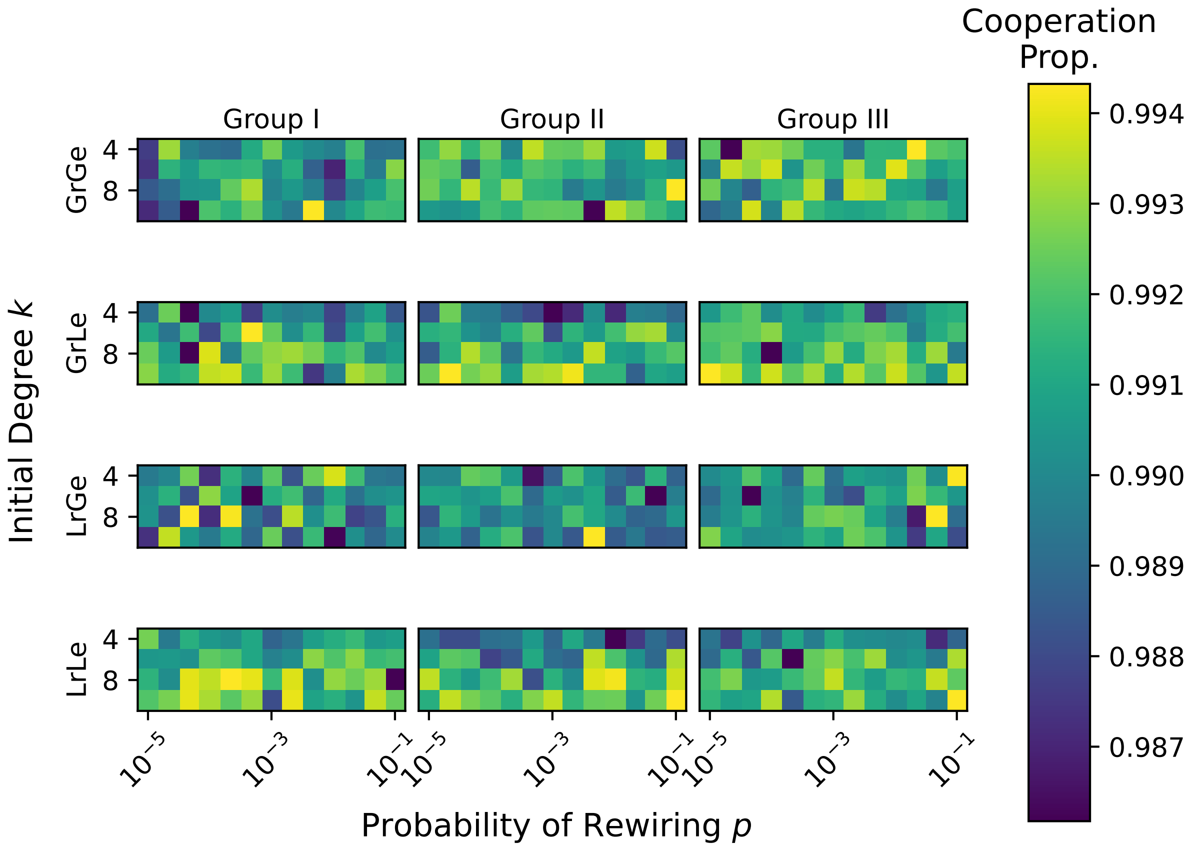

Figure S5. Consistently high levels of cooperation in Small-World networks by initial degree and rewiring probability. Each plot represents the particular combination of reputation and evolution tested (by row) as well as the set of strategies considered (by column). Each data-point represents the average of either 200 (for groups I and III) or 400 (for group II) repeated simulations. The Watts-Strogatz network structure is parameterised by k (initial degree of a ring lattice) and p (rewiring probability for each pair of edges to another previously unconnected node). Simulations culminated in above 98% cooperation across each permutation of parameter and network. There does not appear to be a significant relationship between cooperation and either k or p.

2 Robustness Checks

2.1 Probability of Reputation Transfer against AllD

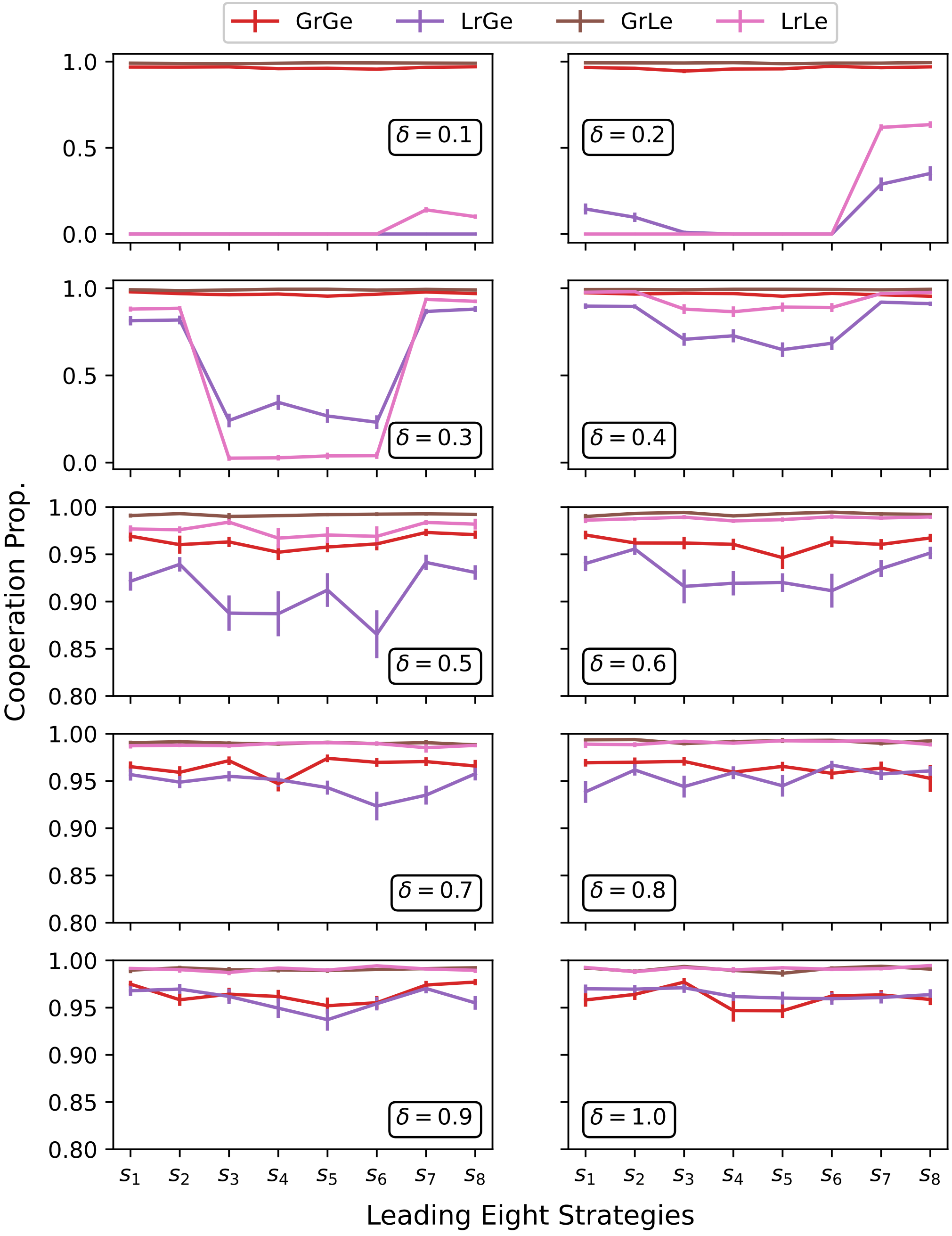

Figure S6. Effect of the probability of reputation transfer on the number of leading strategies. Low δ values provide little to no accurate and up to date reputational information for players, often forcing them to decide their neighbour's reputations at random. Thus we see almost no cooperation amongst populations utilising local information transfer with $\delta = 0.1$. At $\delta = 0.2$ we see that the group III strategies begin to maintain around 50% cooperation. Group I strategies exhibit little to no cooperation here. At $\delta = 0.3$ we see that groups I and III are at almost full strength able to maintain high levels of cooperation within the population. Here for group II, global evolution allows low levels of cooperation while local evolution allows none. When $\delta \geq 0.4$, we see high cooperation with marginal increases in levels with higher δ.

2.2 Local Evolution excess cooperation against probability of reputation transfer

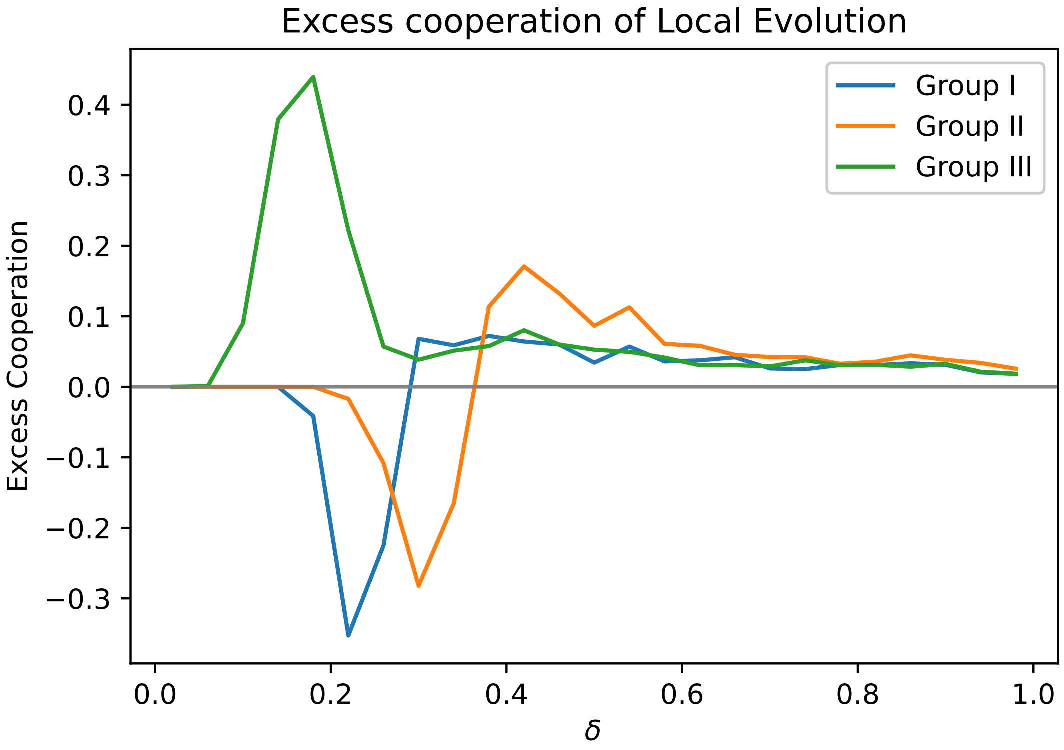

Figure S7. Excess cooperation of local evolution verses global evolution as a function of δ. For group III strategies, local evolution always provides a more beneficial environment for cooperation. For group I and II, while the majority of δ values provide a minimal improvement, global evolution is more advantageous for group I when $\delta \in [0.14, 0.30]$ and when $\delta \in [0.18, 0.38]$ for group II. In summary, if accurate and frequent communication is low, stricter strategies prefer local evolution, while more lenient strategies prefer global evolution.

2.3 Observation error organised by group

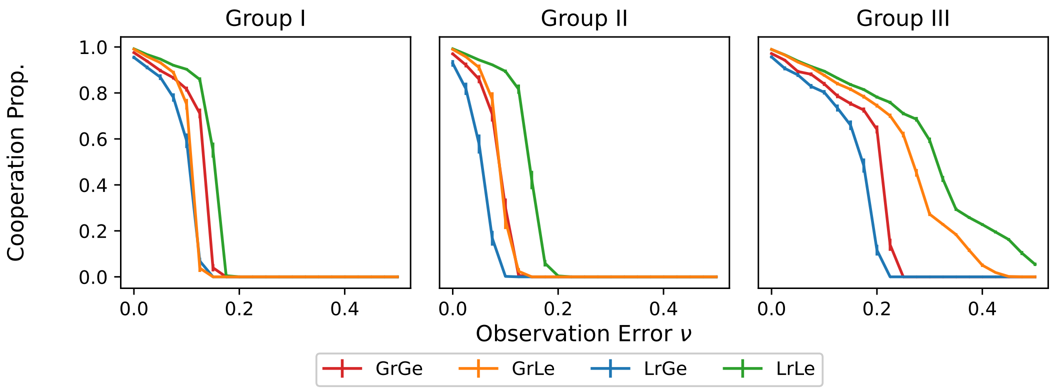

Figure S8. Alternative view of the effect of observation error ν on the proportion of cooperation. Reproduction of Figure 3 comparing the proportion of cooperation by strategy group instead of by reputation and evolution mechanism. The largest benefit of localisation of reputation and evolution occurs for group III strategies. While all groups benefit from this localisation, the differences in minimum ν thresholds are more marginal. In group I, localisation of either reputation or evolution actually harms cooperation compared to the baseline of global reputation and evolution. For group II, local evolution (and global reputation) has little effect from global evolution, while local reputation (and global evolution) harms cooperation.

2.4 The Leading Eight against AllC

2.4.1 Probability of reputation transfer against AllC

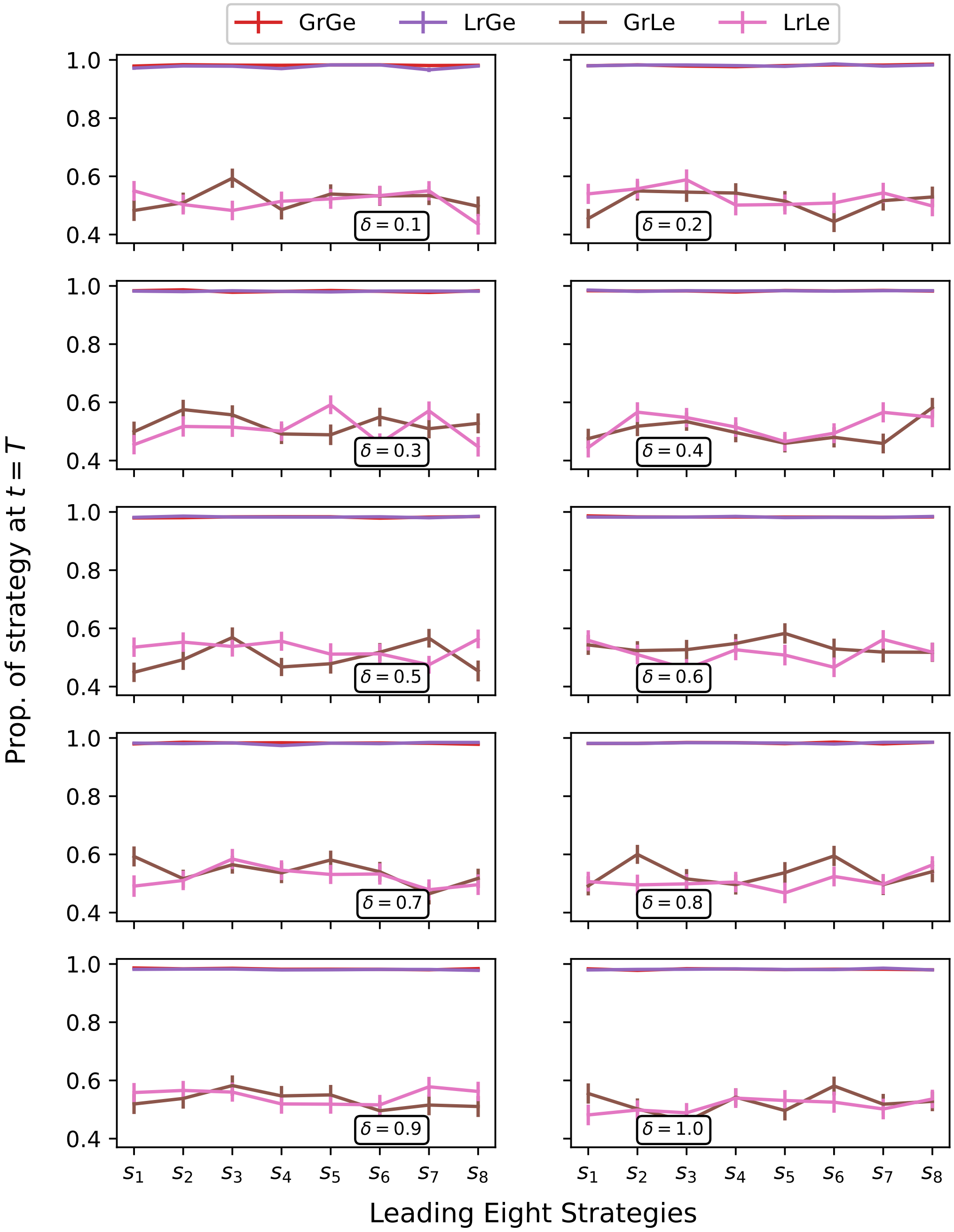

Figure S9. The impact of reputation broadcast probability δ on the final proportion of the leading eight strategies against AllC. Under local evolution, independent of δ, we see that equilibrium is reached with both the leading strategy, and unconditional cooperators in equal proportions within the population. δ has no meaning with global reputation models.

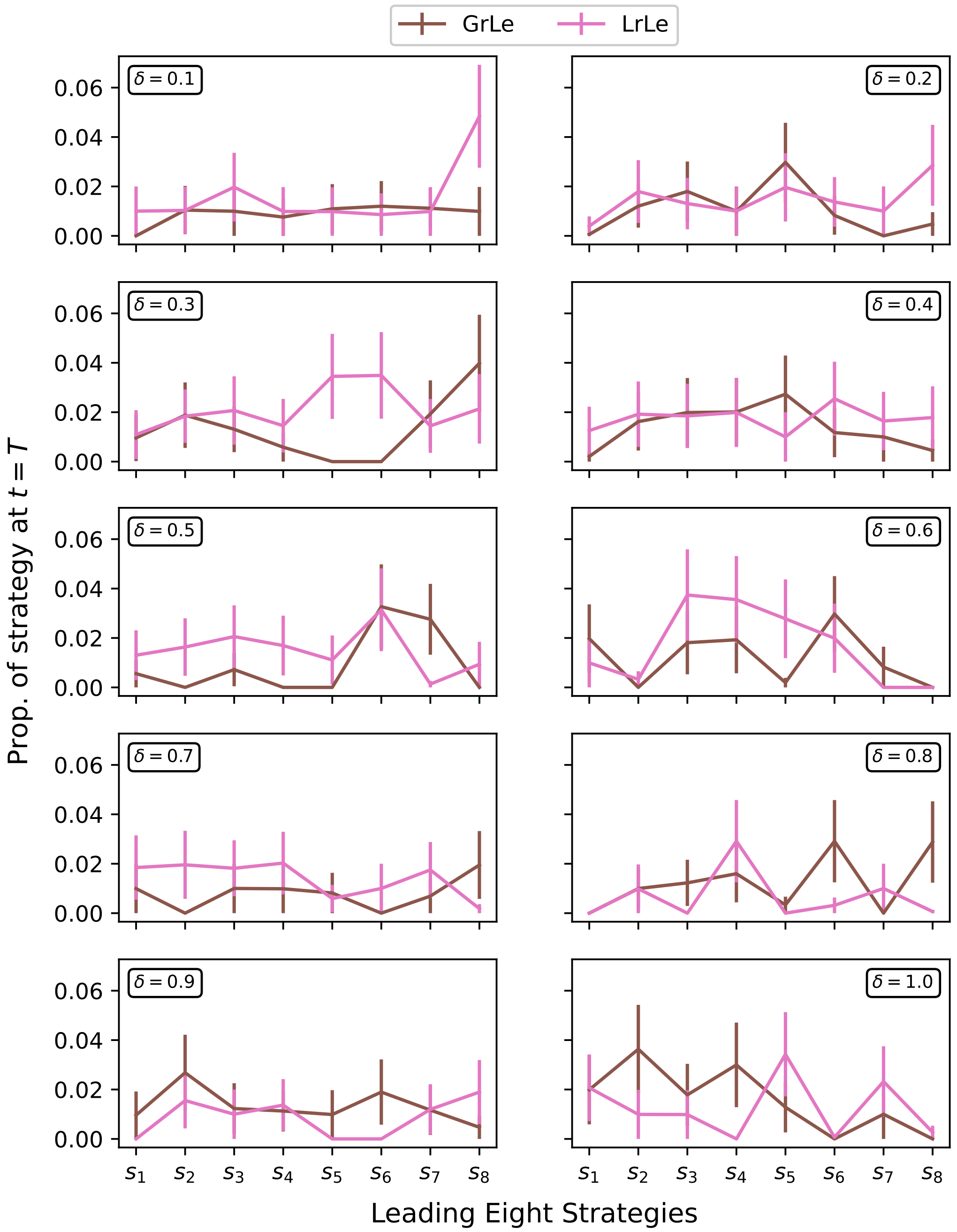

Figure S10. The impact of reputation broadcast probability δ on the final proportion of the leading eight strategies against AllC with $T = 20000$. Here we see that with constant one-sided addition of AllC into the population (through mutation), the equilibrium of AllC and the leading eight strategies eventually break down given enough time and AllC come to dominate.

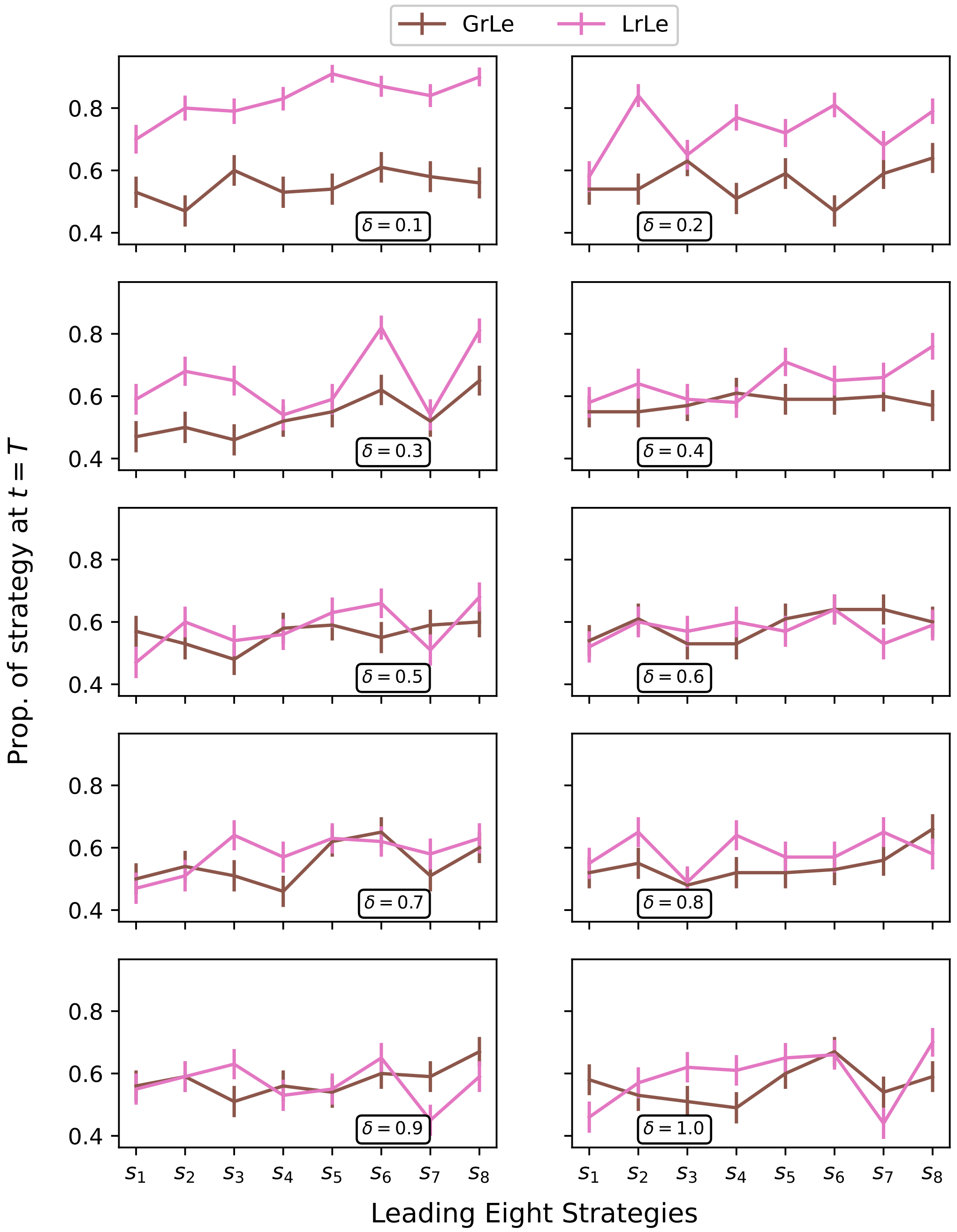

Figure S11. The impact of reputation broadcast probability δ on the final proportion of the leading eight strategies against AllC with $T = 20000$, $\beta = 0$, and initial proportion of AllC set to 50%. With no mutation, the differences between AllC and the leading eight, in a largely cooperative world, become marginal leading to more or less neutral drift between the strategies.

2.4.2 *Speed of strategy update with AllC*

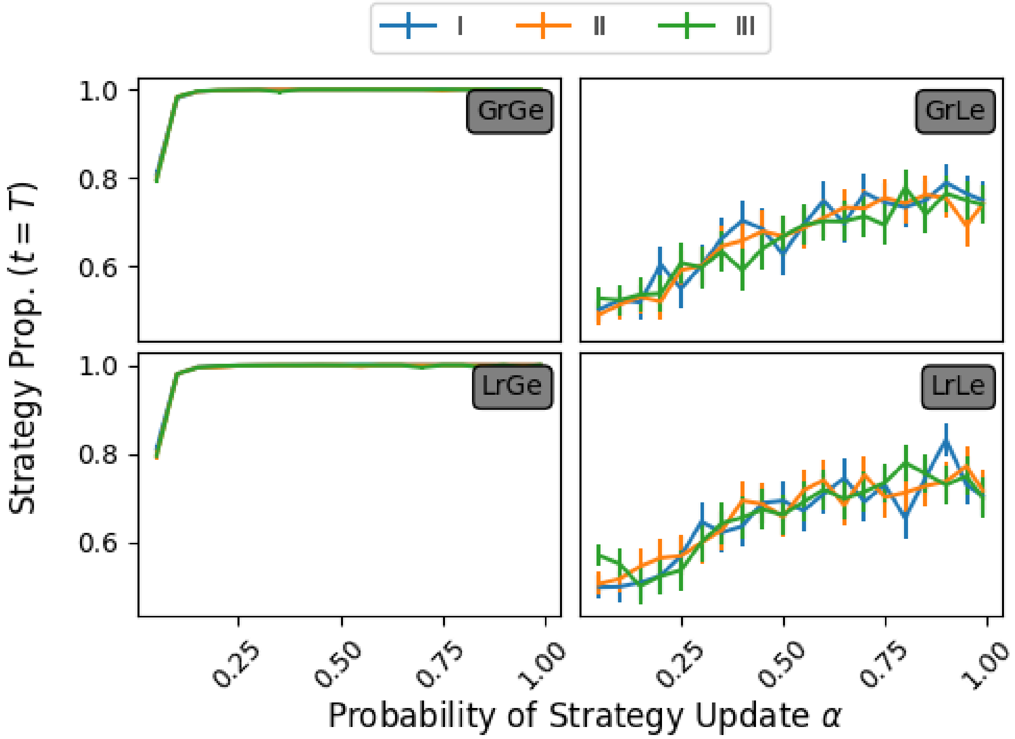

Figure S12. Speed of strategy update of the leading eight against AllC on population state. Mostly independent to α, global learning is superior to Local learning in the dominance of the leading eight over unconditional cooperators. Meanwhile, we see that as α increases, so does the proportion of the leading eight strategy in the population at $t = T$. There seems to be no differentiating behaviour between global and local reputation mechanisms.

2.4.3 AllC cannot invade the leading eight with local learning

As the analysis of robustness against an AllC invasion might modify the relative performance evaluation of the leading eight norms, we run additional analyses similar to Figures 2b and 2a in Supplementary Figures S9 - S12. As expected with global evolution of strategies, AllC is systematically wiped out from the population (Supplementary Figure S9), in line with the leading eight literature.

However, the same cannot be said for local evolution. As both strategies tend to cooperate, they are indistinguishable within the population and thus the relative movement between them is very low leading to an equilibrium (Figure S9). Coupled with a non-zero mutation rate regularly introducing AllC into the population, the leading eight strategies are inevitably invaded (Figure S10). We illustrate this in Figure S11, where we repeat the same experiment but with no ongoing mutation, and populations begin with equal numbers of AllC and leading eight strategists. This results in very little difference from the starting population composition even given a longer simulation time.

2.5 Extreme Mutation Rate

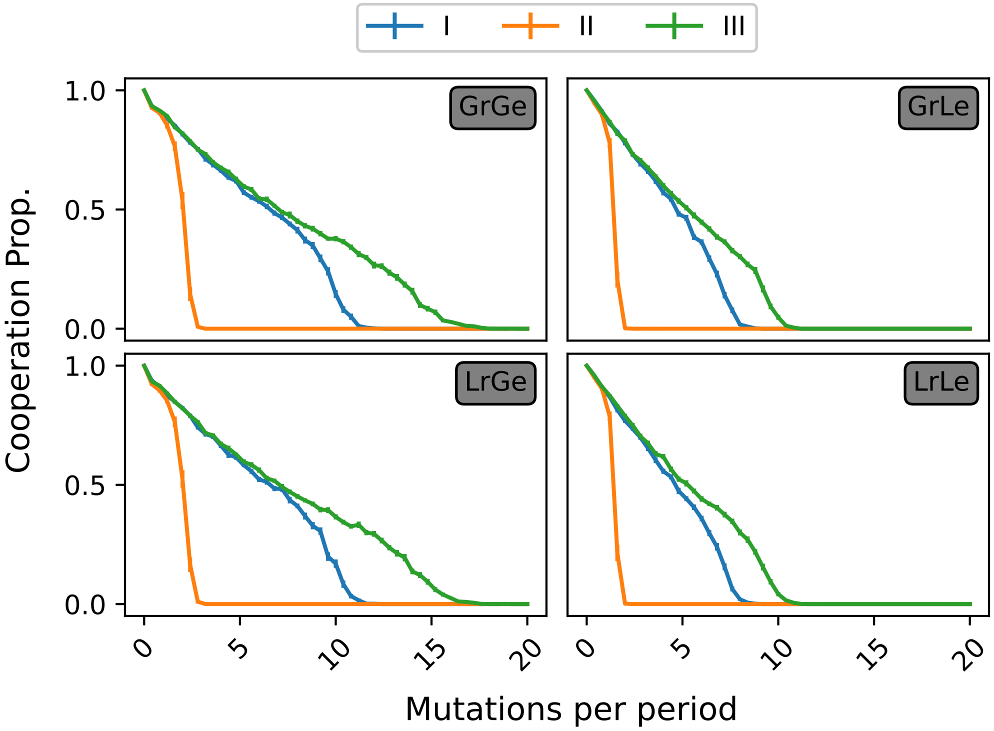

Figure S13. Effect of mutation rate on cooperation. In the extreme case of multiple AllD mutants introduced to the population every period, in line with our findings, we see a clear distinction between the three groups of strategies. On the whole, group II is the weakest. Between groups I and III, the latter is more resilient against invasion by AllD. The choice between global or local reputation makes little difference in this particular case given $\delta = 1$ resulting in perfect information. The choice of local evolution weakens each strategy's resilience towards AllD. Each strategy withstands a lower mutation rate before being complete invasion when compared to global evolution.

2.6 Simulation Length

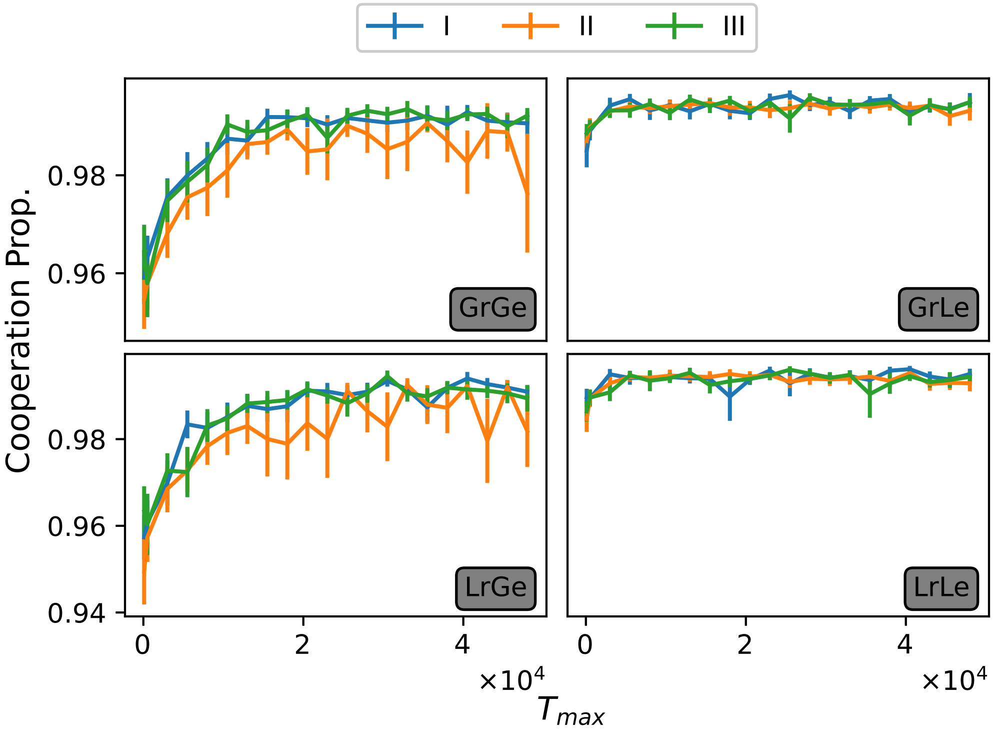

Figure S14. Effect of simulation length on cooperation. The longer the system is allowed to continue (up to a point), the greater the proportion of cooperative actions within the populations. This holds true in both globally evolving and locally evolving networks. However, the former appears to converge at a rate slower than the latter. After 20000 time-steps, the globally evolving populations reach a stable level of cooperation, whereas a higher and less variable level of cooperation is reached between $500 - 3000$ timesteps in locally evolving populations.

2.7 Size of network

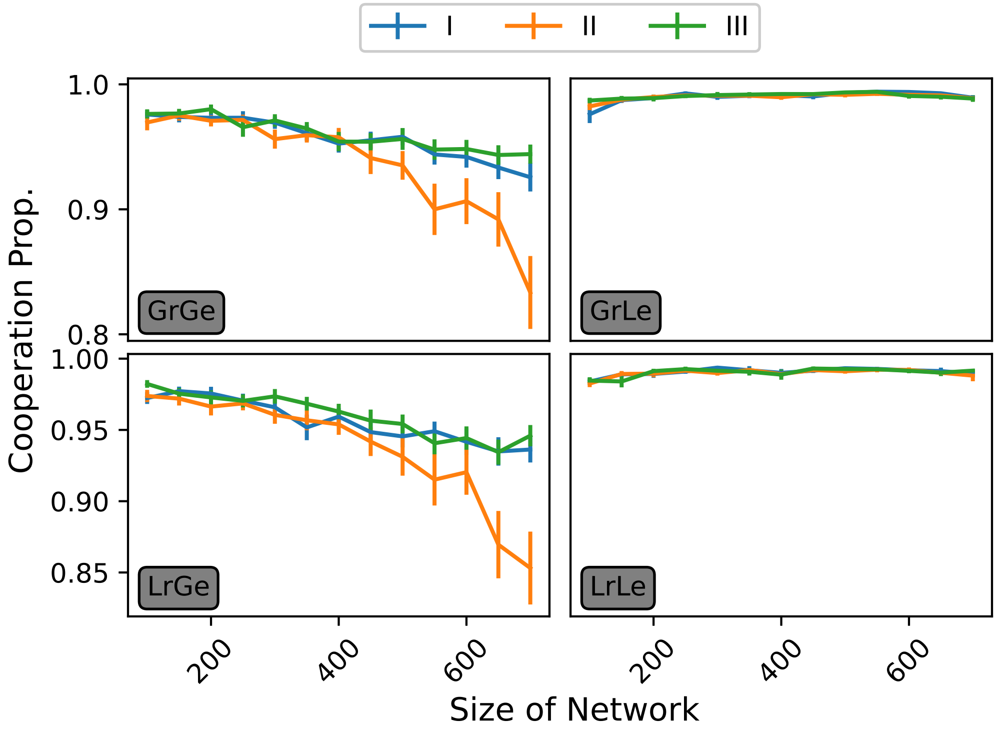

Figure S15. Effect of the size of the network on cooperation. Within the GrGe and LrGe models, we see that as the size of the network increases, the level of cooperation within the population decreases. This is a result of the probability of further interactions within a time-step staying constant as the population size increases, leading to fewer interacting players in each time-step. This allows AllD to spread more quickly as there are simply not enough cooperative interactions amongst the players to compete against the singular but more impactful interactions of the free-riding agents. This is true more so for group II than it is for groups I or III. Under local evolution, it is only the size of the immediate neighbourhood that is relevant for a player's evolution. Thus, cooperation remains largely indifferent to the size of the overall population.

3 Success of groups I and III in AllD populations

Consider momentarily removing ongoing mutation from the simulations. Fig. 4b investigates the effect of cooperation within the network as we vary the initial proportion of AllD agents in the population. Despite extreme levels of unconditional defectors in the population, we find that group I and III strategies mostly emerge dominant whereas group II succumbs much more easily.

To illustrate why, suppose for a single strategy s, we simulate an initial population of 90% unconditional defectors. In this scenario, the vast majority of interactions will be occurring between AllD agents. Under the group I and III norm $d_{00D} = 0$, it is impossible for an unconditional defector to get a good reputation while group II is much more lenient with $d_{00D} = 1$ where mutual defection by disreputable persons can reward both parties with a good reputation. The small number of s strategists rapidly recognise the presence of bad players in the population and will refuse to cooperate with them. This is because the majority of interactions occur between AllD players, awarding them with a bad reputation which causes good s strategists to defect ($p_{10} = D$) and bad s strategists to cooperate in group I ($p_{00} = C$) or defect in groups II and III ($p_{00} = D$).

Therefore the only interactions in which cooperation occurs, is when s players interact amongst themselves. All we require is just a few cooperative and mutually beneficial interactions between agents to make their strategy more lucrative compared to AllD which will have very few profitable actions. We know there will be very few beneficial interactions for AllD as immediately following their first defection with someone good, they will be labelled as someone who should not be helped. From that point on, all agents will refuse to cooperate with them and due to the initial overcrowding of AllD strategists, they will receive no benefit but will interact repeatedly many times drastically reducing the "bargaining power" of their rare payoffs,

$$\text{Average payoff of AllD} = \frac{\text{Low Total Payoff}}{\text{Many AllD Interactions}}$$

in global evolutionary setups. Local evolution (which uses the copy-the-best update rule) restricts the subset of rational players that may decide AllD is more profitable, but also makes the payoff more enticing, relative to the payoff of the s strategists in the immediate neighbourhood leading to agents quickly adopting AllD, successfully defecting once or twice, being branded as someone not worthy of help, and then eventually losing out to the s strategists.

Conversely, if instead the "bargaining power" of a stricter strategy in groups I and III is considered, we see that global evolution allows very rapid growth of the cooperative strategy in the population. This is because even a single positive payoff amongst two players can be seen as the "holy grail" by all defectors in the population causing them to update their strategy with probability α. This is in contrast to locally evolving systems which depend much more on the gradual transformation of non-cooperative to cooperative strategies, moving from neighbourhood to neighbourhood until it becomes dominant. This explains the extra "tail" in the local evolution panel of Fig. 4b which is absent in the global evolution panel. This simply suggests that a longer simulation length would lead to an identical result. This behaviour would also explain the sudden drop in cooperation of globally evolving populations when faced with a very low ongoing mutation in models GrGe and LrGe in Fig. 4a compared with the more gradual decrease as seen in models GrLe and LrLe .

Table of Parameters

Figure	Experiment	Strategies	Init. Prop.	Benefit	Cost	Size	Ω	Max Periods	α	δ	ν	β	ER	RRL	SF	WSSW
2a	Probability of Strategy Update	$s_1 - s_8$	1	2	1	300	0.99	2000	*	1	0	0.1	0.038	nan	nan	nan
2b (Lower)	Probability of Reputation Broadcast	$s_1 - s_8$	1	2	1	300	0.99	2000	0.1	*	0	0.1	0.038	nan	nan	nan
2b (Upper)	Probability of Reputation Broadcast	$s_1 - s_8$	1	2	1	300	0.99	2000	0.1	0.3	0	0.1	0.038	nan	nan	nan
3	Likelihood of observation error	$s_1 - s_8$	1	2	1	300	0.99	3000	0.1	0.6	*	0.1	0.038	nan	nan	nan
4a	Mutation Rate (Micro)	$s_1 - s_8$	1	2	1	300	0.99	3000	0.1	1	0	*	0.038	nan	nan	nan
4b	Initial Proportion of AllD	$s_1 - s_8$	*	2	1	300	0.99	3000	0.1	1	0	0	0.038	nan	nan	nan
S1	Erdös Rényi Density	$s_1 - s_8$	1	2	1	300	0.99	2000	0.1	1	0	0.1	*	nan	nan	nan
S2	Heterogeneity (Macro)	$s_1 - s_8$	1	2	1	300	0.99	3000	0.1	1	0	0.1	*	*	nan	nan
S3	Heterogeneity (Micro)	$s_1 - s_8$	1	2	1	300	0.99	3000	0.1	1	0	0.1	*	*	nan	nan
S4	Barabási-Albert Preferential Attachment	$s_1 - s_8$	1	2	1	300	0.99	2000	0.1	1	0	0.1	nan	nan	*	nan
S5	Watts-Strogatz Small World Parameters	$s_1 - s_8$	1	2	1	300	0.99	2000	0.1	1	0	0.1	nan	nan	nan	*
S6	Probability of Reputation Broadcast	$s_1 - s_8$	1	2	1	300	0.99	2000	0.1	*	0	0.1	0.038	nan	nan	nan
S7	Alt Probability of Reputation Broadcast	$s_1 - s_8$	1	2	1	300	0.99	2000	0.1	0.3	0	0.1	0.038	nan	nan	nan
S8	Alt Likelihood of observation error	$s_1 - s_8$	1	2	1	300	0.99	3000	0.1	0.6	*	0.1	0.038	nan	nan	nan
S9	Prob. Reputation Broadcast AllC	$s_1 - s_8$	1	2	1	300	0.99	2000	0.1	*	0	0.1	0.038	nan	nan	nan
S10	Prob. Reputation Broadcast AllC	$s_1 - s_8$	1	2	1	300	0.99	20000	0.1	*	0	0.1	0.038	nan	nan	nan
S11	Prob. Reputation Broadcast AllC	$s_1 - s_8$	0.5	2	1	300	0.99	20000	0.1	*	0	0	0.038	nan	nan	nan
S12	Probability of Strategy Update AllC	$s_1 - s_8$	1	2	1	300	0.99	2000	*	1	0	0.1	0.038	nan	nan	nan
S13	Mutation Rate (Macro)	$s_1 - s_8$	1	2	1	300	0.99	3000	0.1	1	0	*	0.038	nan	nan	nan
S14	Simulation Length	$s_1 - s_8$	1	2	1	300	0.99	*	0.1	1	0	0.1	0.038	nan	nan	nan
S15	Network Size	$s_1 - s_8$	1	2	1	*	0.99	2000	0.1	1	0	0.1	*	nan	nan	nan

Table S1. Summary of the parameters of each experiment described in this paper. An asterisk * declares the variable that is being investigated in that experiment. Ω represents the probability of further interactions within any single time-step, α represents the probability of strategy update, δ is the probability of reputation transfer, ν is the probability of observation error, β is the expected number of mutants in any given time-step (so $\beta = 0.1$ refers to a single mutant every ten time-steps on average). Under network parameters, ER represents the density of the Erdös Rényi random network, RRL represents the degree d of the lattice, SF refers to the preferential attachment parameter m of the Barabási-Albert model, and WSSW refers to the pair of parameters k, p which represent the initial degree of the ring lattice, and the rewiring probability respectively. Datapoints in each figure represent the mean and error of 100 repeats of each parameter set.
